# Structural and genomic evolutionary dynamics of Omicron variant of SARS-CoV-2 circulating in Madhya Pradesh, India

**DOI:** 10.3389/fmed.2024.1416006

**Published:** 2024-09-11

**Authors:** Suman Dhankher, Pooja Yadav, Shashi Sharma, Ekta Gupta, Ram Govind Yadav, Paban Kumar Dash, Manmohan Parida

**Affiliations:** Virology Division, Defence Research and Development Establishment, Gwalior, India

**Keywords:** BA.2, Lineage, NGS, COVID-19, Genome, Epidemiology

## Abstract

The SARS-CoV-2 Omicron (B.1.1.529) variant emerged in early November 2021 and its rapid spread created fear worldwide. This was attributed to its increased infectivity and escaping immune mechanisms. The spike protein of Omicron has more mutations (>30) than any other previous variants and was declared as the variant of concern (VOC) by the WHO. The concern among the scientific community was huge about this variant, and a piece of updated information on circulating viral strains is important in order to better understand the epidemiology, virus pathogenicity, transmission, therapeutic interventions, and vaccine development. A total of 710 samples were processed for sequencing and identification up to a resolution of sub-lineage. The sequence analysis revealed Omicron variant with distribution as follows: B.1.1, B.1.1.529, BA.1, BA.2, BA.2.10, BA.2.10.1, BA.2.23, BA.2.37, BA.2.38, BA.2.43, BA.2.74, BA.2.75, BA.2.76, and BA.4 sub-lineages. There is a shift noted in circulating lineage from BA.1 to BA.2 to BA.4 over a period from January to September 2022. Multiple signature mutations were identified in S protein T376A, D405N, and R408S mutations, which were new and common to all BA.2 variants. Additionally, R346T was seen in emerging BA.2.74 and BA.2.76 variants. The emerging BA.4 retained the common T376A, D405N, and R408S mutations of BA.2 along with a new mutation F486V. The samples sequenced were from different districts of Madhya Pradesh and showed a predominance of BA.2 and its variants circulating in this region. The current study identified circulation of BA.1 and BA.1.1 variants during initial phase. The predominant Delta strain of the second wave has been replaced by the Omicron variant in this region over a period of time. This study successfully deciphers the dynamics of the emergence and replacement of various sub-lineages of SARS-CoV-2 in central India on real real-time basis.

## Introduction

Globally, as of 24 May 2023, WHO has reported 766,895,075 COVID-19 confirmed cases, which includes 6,935,889 deaths with a total of 13,354,202,412 administered vaccine doses as of 22 May 2023 (1). In spite of global vaccination against COVID-19, the SARS-CoV-2 variants and sub-variants continue to emerge suppressing the older variants causing successive waves of infection. RNA viruses, including SARS-CoV-2, have a high tendency to mutate, and maximum mutations occur in the spike region of the genome, which is responsible for better adaption of the virus into the host system (2). These frequent mutations at the binding sites cause changes in the infectivity and pathogenesis of the disease pattern (3, 4). Omicron emerged as the variant of concern at the end of the year 2021 having several mutations in spike protein and becoming the dominant variant globally by February 2022 (5, 6). Since then, Omicron variants have developed new mutations, giving rise to new sub-variants, with the variants circulating BQ.1, BQ.1.1, and XBB labeled as variants of concern (VOC) by the WHO (2022). In mid-2022, the recombination of BM.1.1.1 (a progeny of BA.2.75) and BJ.1 has contributed to the emergence of the XBB variant (7). Globally spreading XBB sub-variants have substitutions in S protein (e.g., L452R and N460K) causing immune evasion and making it more efficient to spread. In addition, other sets of mutations carried by XBB are V83A, H146Q, Q183E, V445P, and F490S. In February 2023, the Omicron XBB variant (e.g., XBB.1.5 and XBB.1.9) with F486P substitution in the spike (S) protein was predominant (8, 9). March 2023 showed an emergence of XBB.1.16, XBB subvariant with two substitutions in the S protein (E180V and T478R) compared to XBB.1.5 and was labeled as variant under monitoring (VuMs) followed by a variant of interest (VoI) due to its global spread (10–12). XBB.1.5 and XBB.1.16, the two VOIs along with their seven lineages XBB, XBB.1.9.2, BA.2.75, CH.1.1, BQ.1, XBB.1.9.1, and XBB.2.3 as VuMs, are being currently under monitoring by the WHO (12). The maximum mutations in these sub-variants occur in the spike region, that is the receptor-binding domain (RBD), and each mutation in the spike region has contributed to the immune evasion of the host defense and the increased ability of binding ACE-receptor to the XBB spike protein (13–16). XBB.1.16 has been shown to exhibit profound immune evasion and higher growth advantage in the host population (10, 17). Theoretically, these recombinant variants may have slightly different biological characteristics compared to the original strains, which include a different transmission rate, disease outcomes, and/or immune evasion (17–20). As these assumptions are solely based on bioinformatics analysis, *in vitro* and *in vivo* validation is required for further understanding of the effects of these mutations on disease patterns. The concern among the scientific community was huge about this variant, and a piece of updated information on circulating viral strains is important in order to better understand the epidemiology, virus pathogenicity, transmission, therapeutic interventions, and vaccine development. Continuous genomics surveillance is the need of the hour to track the newer emerging variants, sub-variants, and recombinants circulating in the host and successive waves caused by these variants. This would help health authorities in timely preparation to fight against other public health crises. Therefore, in this study, a Next Generation Sequencing Platform employing Oxford Nanopore Based Sequencing Technology was successfully established for whole genome sequencing of SARS-CoV-2. More than 600 samples were processed for sequencing and identification up to a resolution of sub-lineage.

## Materials and methods

### Ethical relevance

The study protocol was approved by DRDE-Institutional Biosafety Committee vide No. IBSC/VIRO-02/2020/PKD. Experiments in this study were conducted according to biosafety and regulatory guidelines. Ethical clearance was obtained from Vidhya Ethics Clearance vide No. VCH/VEC/Feb-2023/01.

### Patients and samples

**Figure 1:**
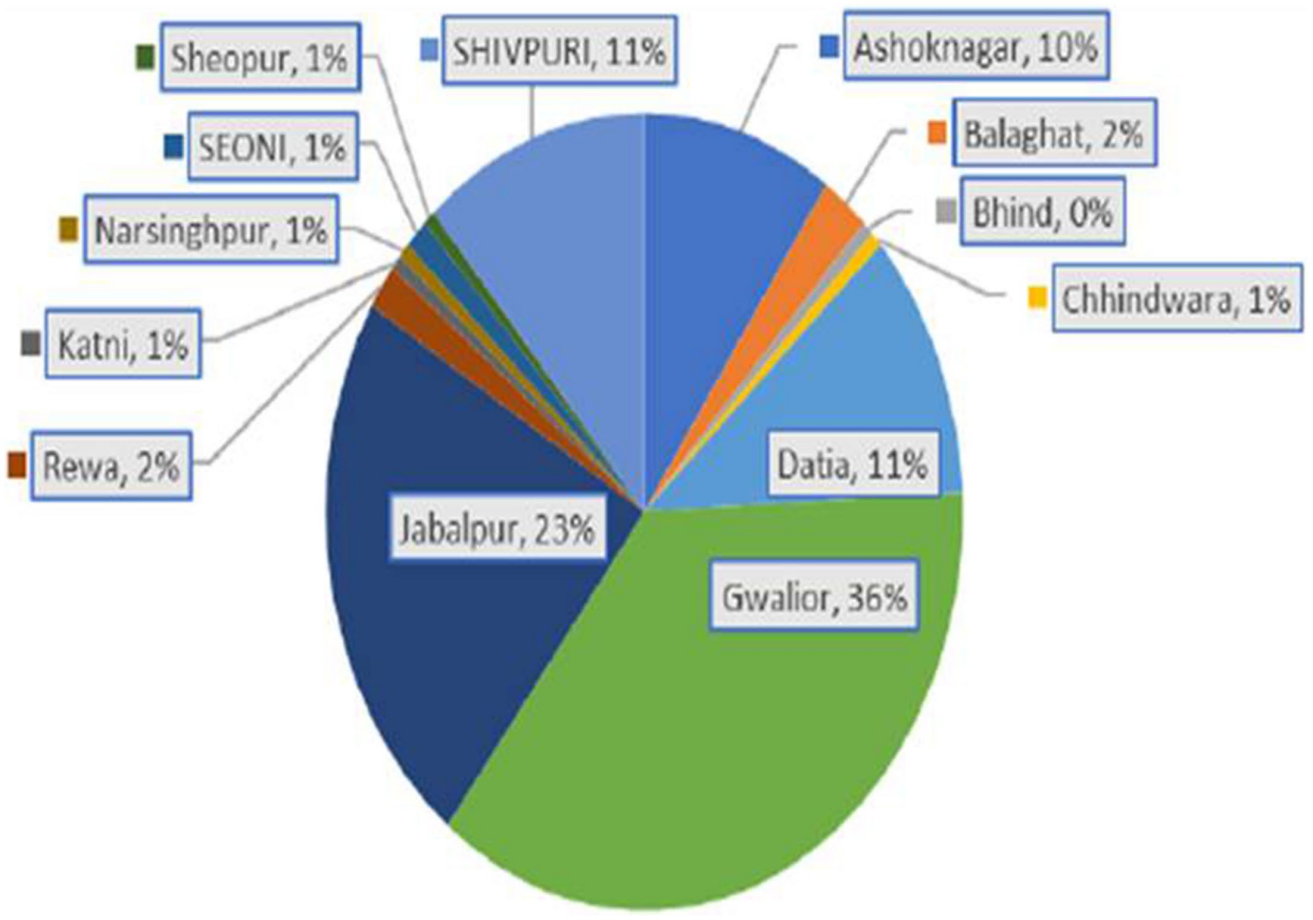
District-wise distribution of sequenced Omicron sub-lineages of SARS-CoV-2.

DRDE-Gwalior as a part of INSACOG-GSL received 710 clinical samples for SARS-CoV-2 genome sequencing from various regions of Madhya Pradesh. The samples studied were naso/oropharyngeal (N/OP) swabs collected in viral transport media and were found to be positive for SARS-CoV-2 infection. A total of 167 samples were sequenced from different regions of Madhya Pradesh during February 2022–May 2023 (Figure 1).

### RNA extraction, real-time RT-PCR, quality assurance, and library preparation

The viral RNA from 710 confirmed SARS-CoV-2 samples received was extracted using a silica column-based QIAamp Viral RNA Mini Kit (Qiagen, Hilden, Germany) according to the instructions of the manufacturer, using 140 μL of the clinical sample. Extracted RNA was eluted using 50 μL of elution buffer. These samples were further processed for screening of the O/N/S gene using qPCR. The SARS-CoV-2 RNA of 167 samples with Ct value of <25 from the samples received was converted to complementary DNA (cDNA) using the Luna One-Step RT-qPCR Kit and amplification through 2 pools of specific primer set was done. The ARTIC protocol’s “midnight” split primer set (pool A and pool B) for SARS-CoV-2 cDNA amplification in two multiplex PCR was used which in single tube 10 μL reaction generates consecutively tiled, non-overlapping 1,200 bp amplicons to avoid overlaps during multiplex PCR (RT-PCR). For each RT-PCR, 8 μL of purified template RNA and 1.5 μL of a 100 M primer pool were used. The reverse transcription step was carried out at 55°C for 30 min, followed by 1 min at 95°C. Then, 34 cycles (pool 1 and pool 2) of denaturation step at 95°C for 20 s followed by annealing and extension in one step at 60°C for 210 s. A final extension was carried out at 65°C. The resulting complementary amplicon mixtures from primer pools 1 and 2 cover nearly the entire SARS-CoV-2 genome. These amplicons were mixed and further taken for library preparation. The manufacturer’s instructions were followed to prepare the library using the Rapid Barcoding Sequencing Kit (SQK-RBK110-96 Oxford Nanopore Technologies, Oxford, United Kingdom) (21, 22).

### Nanopore sequencing

Nanopore sequencing was carried out on an Oxford Nanopore Technologies MinION Mk1B using MinKNOW software. FAST5 and FASTQ files were chosen as output formats. The FASTQ reads generated by MinKNOW were converted to FASTA sequences using COMMANDER software, which is a bioinformatics platform developed by Genotypic Technology Pvt. Ltd. (23). COMMANDER-generated FASTA sequences were subsequently uploaded on the online Nextstrain web app https://clades.nextstrain.org to conduct phylogenetic analyses.

### Detailed genomic characterization and mutation analysis

The amino acid variation for each gene, as well as the net nucleotide and amino acid divergence, was identified using the MEGA software version 7.0.

### Structural analysis of the RBD of Omicron sub-lineages

#### Physicochemical parameter analysis

The ExPASy ProtParam online tool was used to examine the sequences of the Wuhan-Hu-1/2019 (MN908947), Delta, and Omicron (hCoV-19/Japan/TKYS01334/2021, hCoV-19/Botswana/R40B59BHP3321001248/2021, respectively), and Omicron sub-variants (sequenced from this region). Calculations made by ProtParam include the molecular weight (mw), the composition of amino acid, theoretical pI, atomic composition, coefficient of extinction, instability index, half-life predicted, aliphatic index, and the overall hydropathicity of the compound (GRAVY).

### Prediction of spike protein secondary structure and intrinsically unstructured proteins

Wuhan-Hu-1, Delta, and Omicron sub-variants (sequenced from this region) secondary structures were predicted using GOR IV. Information theory and Bayesian statistics are used by the Garnier–Osguthorpe–Robson (GOR) tool to examine secondary protein structure. Using GOR, several sequence alignments are combined in order to learn more and improve secondary structure distinction.

Regions with intrinsic disorder are dynamic conformational ensembles that do not develop a stable three-dimensional structure in physiological situations. The (PONDR^®^ VLXT) predictor of naturally disordered areas was used to forecast the sequences for the Wuhan-Hu-1, Delta variation, and Omicron sub-variants (sequenced from this region) (PONDR).

### Protein stability prediction using I-Mutant 3.0, SIFT, and PolyPhen-2

I-Mutant 3.0 was used to predict the protein stability for sequences of Wuhan-Hu-1, Delta, and Omicron sub-variants. This tool is used for the prediction of single-point changes in protein stability mutations based on support vector machines. It can be used to forecast the protein stability as an indicator.

### SIFT for non-synonymous single nucleotide polymorphisms and protein function prediction

The sorting intolerant from tolerant (SIFT) method is used to determine whether the mutation affects protein function in the wild-type, Delta, and Omicron sub-lineages. SIFT uses amino acid physical characteristics and sequence homology to predict whether an amino acid replacement would influence protein function.

#### PolyPhen-2

This tool predicts amino acid substitution effect on the structure and function by sequence homology, Pfam annotations, and 3D structures from available databases including PDB and tools (ncoils, DSSP, etc.), providing a qualitative prediction and a score [“probably damaging if score > 0.908,” “possibly damaging if value 0.446–0.908,” “benign if value less than equal to 0.446”].

### SARS-CoV-2 RBD-hACE2 docking

Molecular structure from the translated protein of the RBD region was constructed using AlphaFold2 for select Omicron sub-lineages of samples sequenced at this laboratory. Subsequently, protein–protein interaction (SARS-CoV-2 RBD-hACE2) was studied using ClusPro. The available PDB crystal structure of ACE2-SARS-CoV-2 RBD (6M0J) was used, and the docking energies of Delta, Wuhan, and Omicron sub-variants (sequenced from this region) were compared.

## Results

### RNA extraction, real-time RT-PCR, quality assurance, and library preparation

**Figure 2:**
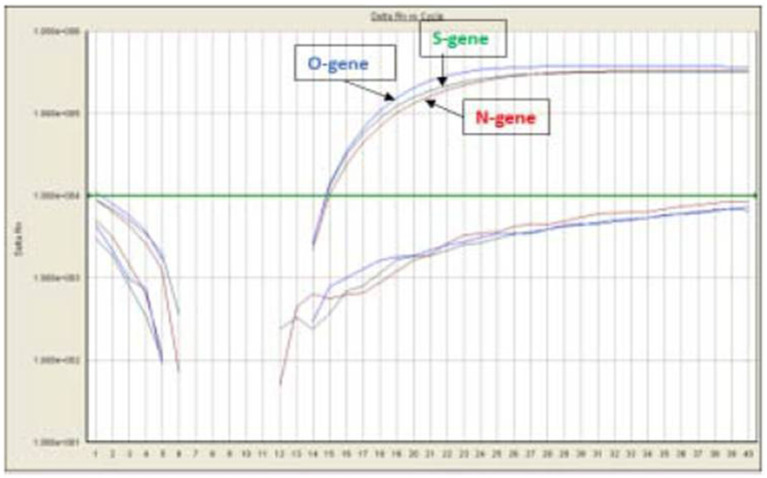
qRT-PCR using SARS-CoV-2 specific primers and probes for the O/N/S gene.

A total of 710 samples (February 2022 to May 2023) positive for SARS-CoV-2 were referred to DRDE, Gwalior, for whole genome sequencing. All samples were screened using real-time RT-PCR using SARS-CoV-2-specific primers and probes for the O/N/S gene exhibiting clear amplification curves for these genes (Figure 2). A total of 167 samples with Ct values of <25 were sequenced in this study. The viral RNA of SARS-CoV-2 of the selected samples was converted to complementary DNA and amplification using 2 pools of specific primers for SARS-CoV-2.

#### Phylogenetic analysis of WGS data

**Figure 3:**
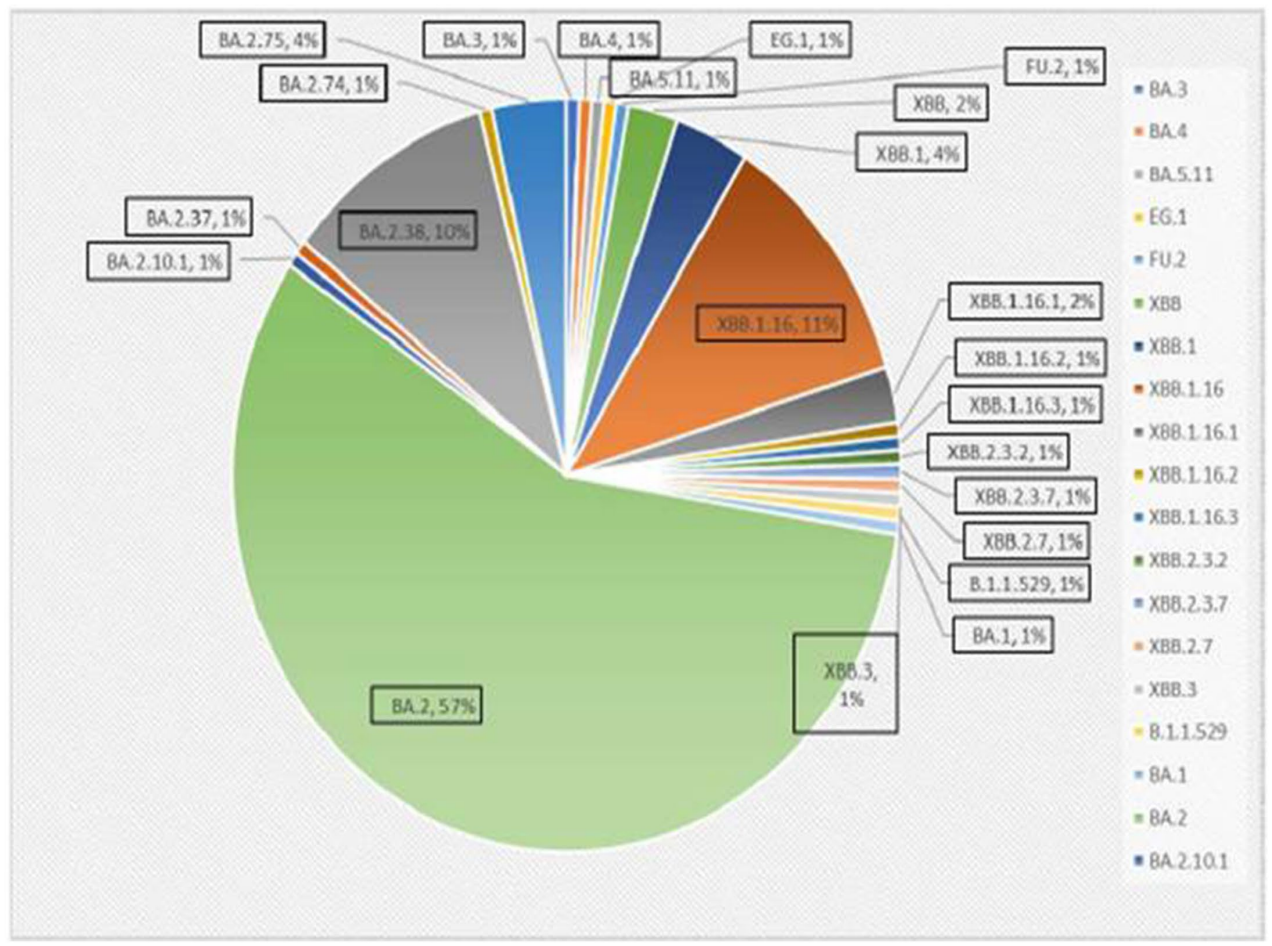
Distribution of Omicron variants in districts of Madhya Pradesh.

**Figure 4:**
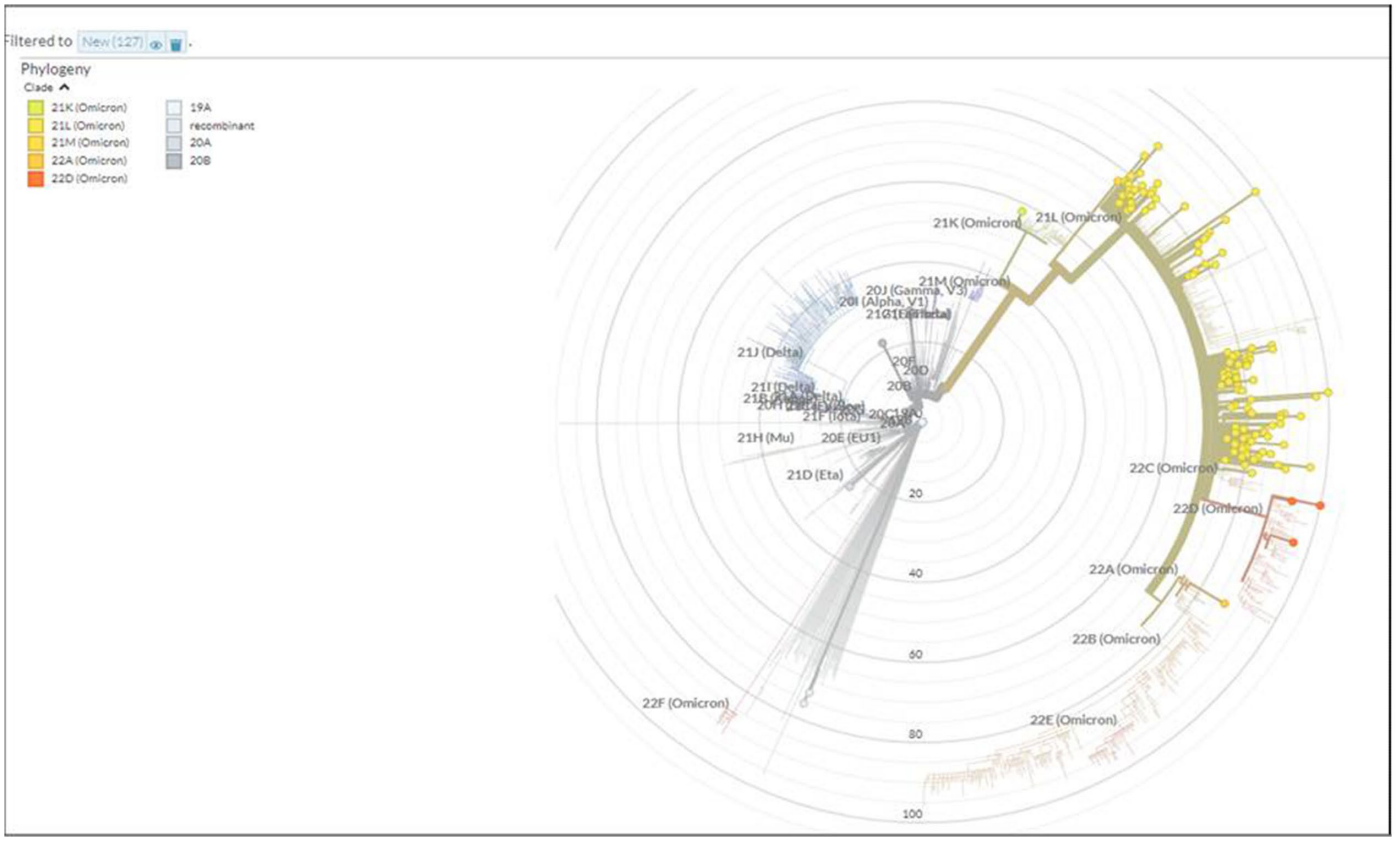
Phylogenetic tree showing the distribution of Omicron variants sequenced in this study.

**Figure 5:**
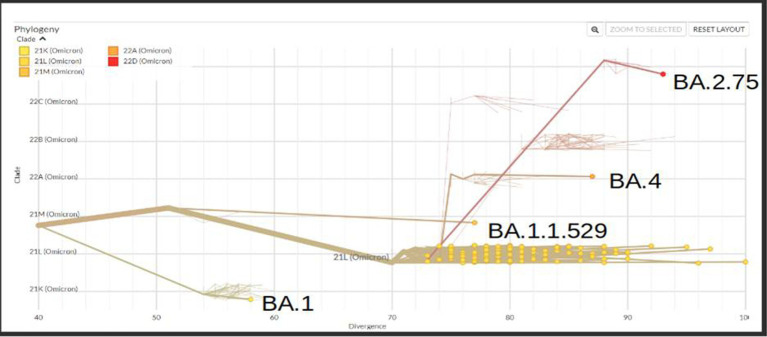
Phylogenetic tree showing the distribution of sequenced Omicron variants in different clades from districts of Madhya Pradesh.

A total of 167 sequences were analyzed for clade classification and mutation calling using Nextcladev2.5.0 against SARS-CoV-2 Reference: Wuhan-Hu-1/2019 (MN908947). Out of 167 samples, 35 showed higher coverage (≥90%) genomes and 132 resulted in partial genomes (127 samples resulting in 70–90% coverage and 05 samples resulting in <70% coverage) and 24 samples remained unassigned. Pango Lineage 4.1.2 (Nextclade) of the sequences belonged to SARS-CoV-2 Omicron sub-lineage and the descendant recombinants with distribution as follows: BA.3 (*n* = 01), BA.4 (*n* = 01), BA.5.11 (*n* = 01), EG.1 (*n* = 01), Fu.2 (*n* = 01), XBB (*n* = 04), XBB.1 (*n* = 06), XBB.1.16 (*n* = 19), XBB.1.16.1 (*n* = 04), XBB.1.16.2 (*n* = 01), XBB.1.16.3 (*n* = 01), XBB.2.3.2 (*n* = 01), XBB.2.3.7 (*n* = 01), XBB.2.7 (*n* = 01), XBB.3 (*n* = 01), unassigned (*n* = 24), B.1.1.529 (*n* = 01), BA.1 (*n* = 01), BA.2 (*n* = 95), BA.2.10.1 (*n* = 01), BA.2.37 (*n* = 01), BA.2.38 (*n* = 17), BA.2.74 (*n* = 01), and BA.2.75 (*n* = 06) lineages (Figure 3). The phylogenetic tree analysis showed Omicron lineages and recombinants with a clear diversion of VOC/VOI (Figure 4). There is a shift in circulating sub-variants of Omicron from BA.1 to BA.2 to BA.4 to XBB over a period of 6 months from 01 January to 30 July 2022. XBB showed predominance thereafter and evolved from XBB.1 to XBB.2 to XBB.3. In February 2023, XBB.1.16 emerged to be causing another peak of infections in the region. The distribution of these sub-variants in clades in the Phylogenetic tree^1^ is shown in Figure 5.

**Figure 6:**
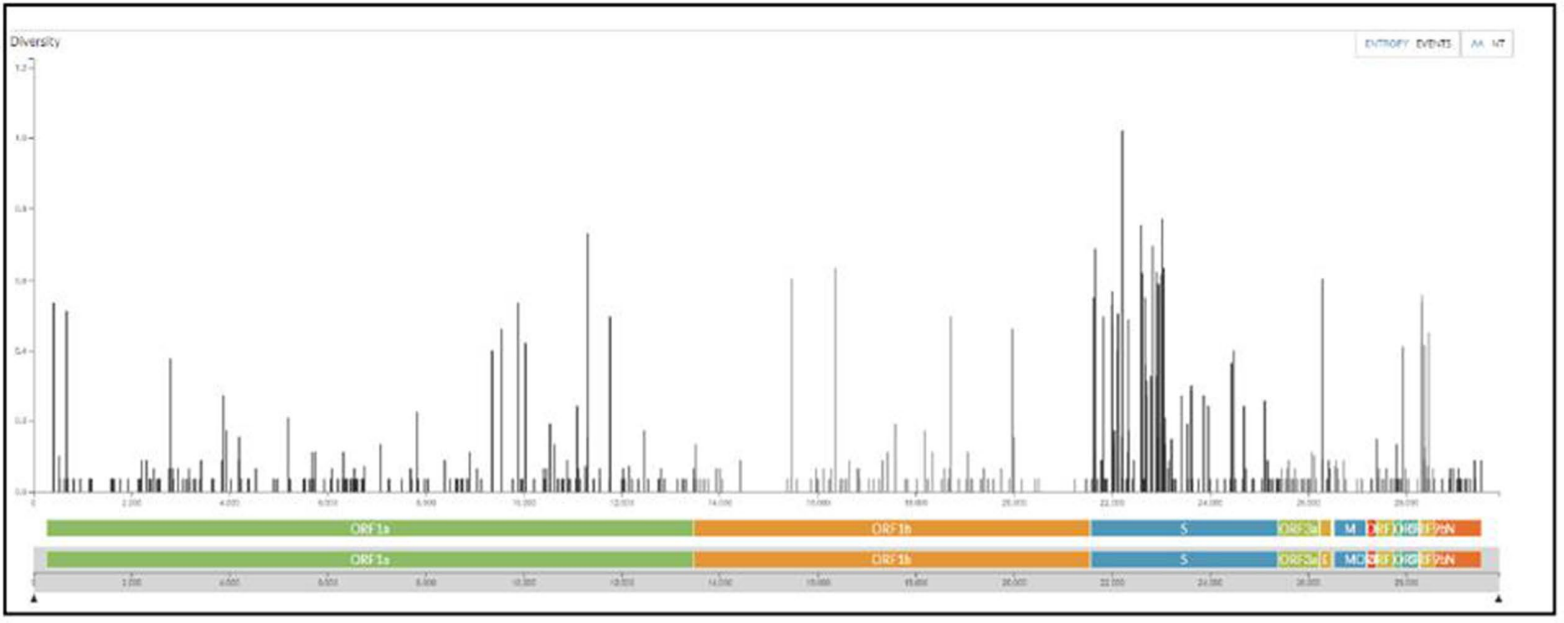
The maximum number of events in AA changes seen in the spike region of Omicron variants sequenced from the regions of Madhya Pradesh.

All samples sequenced were found to be sub-variants of Omicron. The maximum number sequenced was BA.2 sub-variant with events in AA changes in the spike region followed by Orf1a among all (Figure 6).

#### Structural analysis of the RBD of Omicron sub-lineages

Further looking into the physical parameters of the RBD protein of select sequenced Omicron sub-lineages were analyzed using the online ExPASy ProtParam tool to look for the change in composition and stability of this protein over a period of time in various sub-lineages (Table 1). AA in the RBD region of Wuhan-Hu-1, Delta, and Omicron variant is the same, i.e., 223; however, the weight and theoretical pI of Omicron (Wt: 25387.92, pI: 9.17) are higher than the Wuhan-Hu-1 (Wt: 25168.50, pI: 9.09) and the Delta variant (Wt: 25098.40, pI: 8.91). The pH is a protein’s isoelectric point (pI) when the surface is fully charged with its net charge being less than zero. The presence of alkaline proteins is indicated by pI values greater than 7, whereas a number lower than 7 denotes acidic protein. The alkalinity of RBD of Omicron sub-variants is higher than Wuahn-Hu-1. However, there has been a decrease in the weight and alkalinity of the later variants (BA.2.74, BA.5, and recombinants—XBB). XBB.2.3.7 (DRDE-1148) was found to be less alkaline, with a lower weight and stability index (pI: 8.67, Wt: 24947.37, II: 22.70) somewhat similar to Delta variant, and therefore possess a potential to cause serious infections. This variant is one of the VuM declared by the WHO.

**Table 1:** Physical parameters of the RBD protein of Omicron sub-variants, Omicron and Delta with reference to Wuhan-Hu-1.

	pI	Molecular weight (Mw)	Instability index	GRAVY	Aliphatic index
WUHAN_RBD	9.09	25168.50	22.69	−0.259	71.61
DELTA_RBD	8.91	25098.40	21.25	−0.310	69.87
OMICRON_RBD	9.17	25387.92	29.64	−0.253	73.81
1058_RBD(B.1.1)	9.02	25071.39	23.07	−0.267	71.61
1003_RBD(BA.1.5)	9.17	25387.92	29.64	−0.253	73.81
1044_RBD(BA.2)	9.17	25261.77	27.82	−0.226	72.51
1015_RBD(BA.2.10)	9.17	25261.77	27.82	−0.226	72.51
1017_RBD(BA.2.37)	9.17	25261.77	27.82	−0.226	72.51
1092_RBD(BA.2.38)	9.17	25248.77	25.85	−0.214	72.51
1125_RBD(BA.2.74)	9.09	25224.72	31.41	−0.218	70.76
1134_RBD(BA.2.75)	9.24	25299.86	27.32	−0.224	72.51
1129_RBD(BA.4)	9.17	25228.69	27.32	−0.253	72.06
1146_RBD(BA.5.2)	9.09	25173.61	27.88	−0.236	72.06
1142_RBD(XBB)	9.16	25122.56	29.88	−0.262	71.21
1145_RBD(XBB.3)	9.16	25122.56	29.88	−0.262	71.21
1148_RBD(XBB.2.3.7)	8.67	24947.37	22.70	−0.196	72.06
1169_RBD(XBB.1.16)	9.17	25160.61	30.02	−0.269	71.21
1176_RBD(XBB.1)	9.17	25160.61	30.02	−0.269	71.21
1194_RBD(BA.3)	9.17	25096.57	28.78	−0.236	72.96

In the evaluation of the primary structure of Omicron, the AA composition of spike protein shows a shift of increase in charged AA (arginine, lysine, aspartic acid, and glutamic acid), which leads to a greater degree of charged residue exposure by contributing to salt bridge formation (Table 2). Sequenced Omicron variants were found to have a higher content of amino acids such as leucine, phenylalanine, and proline, which are non-polar, and low content of polar amino acids such as asparagine and glutamine. The hydrophobic amino acids are located in the protein core, thus making it solvent inaccessible.

**Table 2:** Amino acid composition comparison between Omicron variant, Omicron sub-variants, and Delta with reference to wild type (Wuhan-Hu-1).

	Wuhan-Hu-1 RBD	Delta-RBD	Omicron-RBD	1058_RBD(B.1.1)	1003_RBD(BA.1.5)	1044_RBD(BA.2)	1015_RBD(BA.2.10)	1017_RBD(BA.2.37)	1092_RBD(BA.2.38)	1125_RBD(BA.2.74)	1134_RBD(BA.2.75)	1129_RBD(BA.4)	1146_RBD(BA.5.2)	1142_RBD(XBB)	1145_RBD(XBB.3)	1148RBD(XBB.2.3.7)	1169 RBD(XBB.1.16)	1176 RBD(XBB.1)	1194 RBD(BA.3)
Ala (A)%	5.4%	5.4%	5.8%	5.40%	5.8%	**6.3%**	**6.3%**	**6.3%**	**6.3%**	**6.3%**	**6.3%**	**6.3%**	**6.3%**	**6.3%**	**6.3%**	5.8%	6.3%	6.3%	6.3%
Arg (R)%	**4.9%**	**5.4%**	**5.8%**	4.90%	**5.8%**	5.4%	5.4%	5.4%	5.4%	4.9%	4.9%	5.4%	4.9%	**4.5%**	**4.5%**	4.00%	4.90%	4.90%	4.90%
Asn (N)%	9.4%	9.4%	9.4%	9.40%	9.4%	**9.9%**	**9.9%**	**9.9%**	9.4%	**9.9%**	9.4%	**9.9%**	**9.9%**	**9.4%**	**9.4%**	9.40%	9.40%	9.40%	9.40%
Asp (D)%	**4.0%**	**4.0%**	4.5%	4.00%	**4.5%**	4.0%	4.0%	4.0%	4.0%	4.0%	3.6%	4.0%	4.0%	**3.6%**	**3.6%**	4.50%	3.60%	3.60%	3.60%
Cys (C)%	4.0%	4.0%	4.0%	4.0%	4.0%	4.0%	4.0%	4.0%	4.0%	4.0%	4.0%	4.0%	4.0%	4.0%	4.0%	4.00%	4.00%	4.00%	4.00%
Gln (Q)%	3.1%	3.1%	**2.2%**	**3.6%**	2.2%	2.2%	2.2%	2.2%	2.2%	2.2%	**2.7%**	**2.7%**	**2.7%**	**2.7%**	**2.7%**	2.70%	2.70%	2.70%	2.70%
Glu (E)%	**3.1%**	**3.1%**	**2.7%**	**2.7%**	**2.7%**	2.7%	2.7%	2.7%	2.7%	2.7%	2.7%	2.7%	2.7%	2.7%	2.7%	2.70%	2.70%	2.70%	2.70%
Gly (G)%	6.7%	6.7%	**5.4%**	6.7%	5.4%	6.3%	6.3%	6.3%	6.3%	6.3%	5.8%	6.3%	6.3%	**5.8%**	**5.8%**	6.30%	5.80%	5.80%	6.30%
His (H)%	0.4%	0.4%	0.9%	0.9%	0.9%	0.9%	0.9%	0.9%	0.9%	0.9%	**1.3%**	0.9%	0.9%	**1.3%**	**1.3%**	0.40%	1.30%	1.30%	0.90%
Ile (I)%	4.0%	4.0%	4.0%	4.0%	4.0%	4.0%	4.0%	4.0%	4.0%	4.0%	4.0%	4.0%	4.0%	**4.5%**	**4.5%**	4.00%	4.50%	4.50%	4.50%
Leu (L)%	6.3%	5.8%	6.7%	6.3%	6.7%	6.3%	6.3%	6.3%	6.3%	**5.8%**	**6.3%**	**5.8%**	**5.8%**	**5.8%**	**5.8%**	6.30%	5.80%	5.80%	6.30%
Lys (K)%	5.4%	5.8%	5.8%	5.4%	**5.8%**	5.8%	5.8%	5.8%	5.8%	5.8%	6.3%	5.8%	5.8%	**6.3%**	**6.3%**	5.40%	5.80%	5.80%	5.80%
Met (M)%	0.0%	0.0%	0.0%	0.0%	0.0%	0.0%	0.0%	0.0%	0.0%	**0.4%**	0.0%	0.0%	0.0%	0.0%	0.0%	0.00%	0.00%	0.00%	0.00%
Phe (F)%	7.2%	7.2%	7.6%	7.2%	7.2%	**8.1%**	**8.1%**	**8.1%**	**8.1%**	**8.1%**	**8.1%**	**7.6%**	**7.6%**	**7.2%**	**7.2%**	7.20%	7.20%	7.20%	7.20%
Pro (P)%	5.8%	5.8%	**6.3%**	**6.3%**	5.8%	**6.3%**	**6.3%**	**6.3%**	**6.3%**	**6.3%**	**6.3%**	**6.3%**	**6.3%**	**6.7%**	**6.7%**	5.40%	7.20%	7.20%	6.70%
Ser (S)%	7.6%	7.6%	**6.7%**	**6.7%**	7.6%	**6.3%**	**6.3%**	**6.3%**	**6.3%**	**6.3%**	**6.7%**	**6.3%**	**6.3%**	**7.6%**	**7.6%**	8.50%	7.20%	7.20%	7.20%
Thr (T)%	5.8%	**5.4%**	**5.4%**	**5.4%**	**5.8%**	**4.9%**	**4.9%**	**4.9%**	**5.4%**	**5.4%**	**4.9%**	**4.9%**	**5.4%**	**5.4%**	**5.4%**	6.30%	5.40%	5.40%	5.40%
Trp (W)%	0.9%	0.9%	0.9%	0.9%	0.9%	0.9%	0.9%	0.9%	0.9%	0.9%	0.9%	0.9%	0.9%	0.9%	0.9%	0.90%	0.90%	0.90%	0.90%
Tyr (Y)%	6.7%	6.7%	6.7%	6.7%	6.3%	6.7%	6.7%	6.7%	6.7%	**6.7%**	**6.7%**	**6.7%**	**6.7%**	**6.7%**	**6.7%**	6.70%	6.70%	6.70%	6.70%
Val (V)%	9.0%	9.0%	9.0%	9.0%	9.0%	9.0%	9.0%	9.0%	9.0%	9.0%	9.0%	**9.4%**	**9.4%**	**8.5%**	**8.5%**	9.00%	8.50%	8.50%	8.50%
Pyl (O)%	0.0%	0.0%	0.0%	0.0%	0.0%	0.0%	0.0%	0.0%	0.0%	0.0%	0.0%	0.0%	0.0%	0.0%	0.0%	0.00%	0.00%		0.00%
Sec (U)%	0.0%	0.0%	0.0%	0.0%	0.0%	0.0%	0.0%	0.0%	0.0%	0.0%	0.0%	0.0%	0.0%	0.0%	0.0%	0.00%	0.00%		0.00%

The increase in the percentage of amino acid composition for Omicron sub-variants in RBD is marked in bold.

There is an evolutionary change in the secondary structure of SARS-CoV-2 (Table 3) with a difference in the alpha-helix structure of Omicron (8.5%) higher than the Delta variant (5.8%) but has less extended strand and the random coil structure. These variants were further subjected to look for the intrinsically disordered areas of viral spike proteins, for this some of the selected sequenced samples with full spike protein coverage were evaluated using PONDR^®^ VLXT (Table 4). These disordered regions are associated with viral infectivity and pathogenicity. A value between 0.2 and 0.5 is flexible, and anticipated disorder scores of more than 0.5 are considered inherently disordered. It was observed the initial strain of Omicron has a less disordered area when compared with the Wuhan-Hu-1 and the Delta variant. However, a reversal of a number of disordered regions in the variants of Omicron BA.2 similar to delta and Wuhan was seen. Subsequently, a difference was noted in the initial segment [409]–[410] of disordered from the Delta and wild type, which was common in these sub-lineages. As T470-F490 loop and Q498-Y505 structure within RBD of SARS-CoV-2 is an important site that interacts with RBD and ACE2 and the prediction ranges from 150 to 157 of residues in ISTEIYQA in Wuhan-Hu-1-RBD, 155–157 of residues EIY in RBD of Delta variant, but no disorder residues were seen in this region of any of the variants of Omicron BA.2 (Table 5). This transition of the disordered segment 150–157 of RBD could be an important factor for the RBD stability and its binding capacity to ACE2 receptors, hence leading to the increased transmissibility of the virus and evasion of host-immune response from neutralizing antibodies. However, the evolving recombinants (XBB.1, XBB.2.3.7, and XBB.1.16) and BA.3 have shown to have an increase in the number of disordered regions similar to 150–159 and 147–168. This transition explains the new peak of infection caused by these variants and the continuing evolving nature of the virus to evade the host response.

**Table 3:** Secondary structure prediction of RBD of SARS-Cov-2 and comparison of Delta, Omicron, and Omicron variants RBD with reference to wild type (Wuhan-Hu-1).

	Wuhan-Hu-1 RBD	Delta-RBD	Omicron-RBD	1058_RBD(B.1.1)	1003_RBD(BA.1.5)	1044_RBD(BA.2)	1015_RBD(BA.2.10)	1017_RBD(BA.2.37)	1092_RBD(BA.2.38)	1125_RBD(BA.2.74)	1134_RBD(BA.2.75)	1129_RBD(BA.4)	1146_RBD(BA.5.2)	1142_RBD(XBB)	1145_RBD(XBB.3)	1148_RBD(XBB.2.3.7)	1169-RBD(XBB.1.16)	1176_RBD(XBB.1)	1194_RBD(BA.3)
Alpha-helix (Hh)	15 (6.7%)	13 (5.8%)	19 (8.5%)	15 (6.73%)	19 (8.5%)	20 (8.97%)	20 (8.97%)	**20 (8.9%)**	**20 (8.9%)**	**5 (2.24%)**	19 (8.5%)	18 (8.07%)	11 (4.93%)	11 (4.93%)	11 (4.93%)	8 (3.59%)	11 (4.93%)	11 (4.93%)	12 (5.8%)
3_10_helix (Gg)	0 (0.0%)	0 (0.0%)	0 (0.0%)	0 (0.0%)	0 (0.0%)	0 (0.0%)	0 (0.0%)	0 (0.0%)	0 (0.0%)	0 (0.0%)	0 (0.0%)	0 (0.0%)	0 (0.0%)	0 (0.0%)	0 (0.0%)	0 (0.0%)	0 (0.0%)	0 (0.0%)	0 (0.0%)
Pi helix (Ii)	0 (0.0%)	0 (0.0%)	0 (0.0%)	0 (0.0%)	0 (0.0%)	0 (0.0%)	0 (0.0%)	0 (0.0%)	0 (0.0%)	0 (0.0%)	0 (0.0%)	0 (0.0%)	0 (0.0%)	0 (0.0%)	0 (0.0%)	0 (0.0%)	0 (0.0%)	0 (0.0%)	0 (0.0%)
Beta bridge (Bb)	0 (0.0%)	0 (0.0%)	0 (0.0%)	0 (0.0%)	0 (0.0%)	0 (0.0%)	0 (0.0%)	0 (0.0%)	0 (0.0%)	0 (0.0%)	0 (0.0%)	0 (0.0%)	0 (0.0%)	0 (0.0%)	0 (0.0%)	0 (0.0%)	0 (0.0%)	0 (0.0%)	0 (0.0%)
Extended strand (Ee)	53 (23.7%)	53 (23.7%)	39 (17.4%)	53 (23.77%)	39 (17.49%)	39 (17.49%)	39 (17.49%)	39 (17.49%)	**41 (18.39%)**	**56 (25.11%)**	39 (17.4%)	39 (17.49%)	46 (20.63%)	51 (22.87%)	51 (22.87%)	68 (30.49%)	51 (22.87%)	51 (22.87%)	46 (20.63%)
Beta turn (Tt)	0 (0.0%)	0 (0.0%)	0 (0.0%)	0 (0.0%)	0 (0.0%)	0 (0.0%)	0 (0.0%)	0 (0.0%)	0 (0.0%)	0 (0.0%)	0 (0.0%)	0 (0.0%)	0 (0.0%)	0 (0.0%)	0 (0.0%)	0 (0.0%)	0 (0.0%)	0 (0.0%)	0 (0.0%)
Bend region (Ss)	0 (0.0%)	0 (0.0%)	0 (0.0%)	0 (0.0%)	0 (0.0%)	0 (0.0%)	0 (0.0%)	0 (0.0%)	0 (0.0%)	0 (0.0%)	0 (0.0%)	0 (0.0%)	0 (0.0%)	0 (0.0%)	0 (0.0%)	0 (0.0%)	0 (0.0%)	0 (0.0%)	0 (0.0%)
Random coil (Cc)	155 (69.5%)	157 (70.4%)	165 (73.9%)	155 (69.51%)	165 (73.99%)	164 (73.54%)	164 (73.54%)	164 (73.54%)	162 (72.65%)	162 (72.6%)	165 (73.9%)	166 (74.44%)	166 (74.44%)	161 (72.2%)	161 (72.2%)	147 (65.92%)	161 (72.20%)	161 (72.20%)	165 (73.99%)
Ambiguous states (?)	0 (0.0%)	0 (0.0%)	0 (0.0%)	0 (0.0%)	0 (0.0%)	0 (0.0%)	0 (0.0%)	0 (0.0%)	0 (0.0%)	0 (0.0%)	0 (0.0%)	0 (0.0%)	0 (0.0%)	0 (0.0%)	0 (0.0%)	0 (0.0%)	0 (0.0%)	0 (0.0%)	0 (0.0%)
Other states	0 (0.0%)	0 (0.0%)	0 (0.0%)	0 (0.0%)	0 (0.0%)	0 (0.0%)	0 (0.0%)	0 (0.0%)	0 (0.0%)	0 (0.0%)	0 (0.0%)	0 (0.0%)	0 (0.0%)	0 (0.0%)	0 (0.0%)	0 (0.0%)	0 (0.0%)	0 (0.0%)	0 (0.0%)

The increase in the percentage of secondary structure for various sub-lineages is marked in bold.

**Table 4:** Intrinsically disordered prediction for Omicron variant of the spike region using PONDR^®^ VLXT.

Variant	No. of residues disordered	Overall percent disordered	Predicted disorder segment	Number disordered regions
Wuhan-HU-1	98	7.70%	[17]–[20] [468]–[475] [601]–[608] [672]–[709] [869]–[871] [945]–[950] [982]–[986] [992]–[994] [1023]–[1023] [1174]–[1194] [1264]–[1264]	11
DELTA_SPIKE	101	7.93%	[471]–[473] [601]–[610] [674]–[709] [869]–[871] [940]–[957] [982]–[986] [992]–[994] [1023]–[1023] [1174]–[1194] [1264]–[1264]	10
OMICRON_SPIKE	89	6.99%	[17]–[20] [601]–[610] [678]–[709] [870]–[871] [978]–[995] [1023]–[1023] [1174]–[1194] [1264]–[1264]	8
1090_BA.2	88	6.91%	[409]–[410] [601]–[610] [678]–[709] [869]–[871] [945]–[950] [982]–[986] [992]–[994] [1023]–[1023] [1174]–[1194] [1261]–[1265]	10

**Table 5:** Intrinsically disordered prediction for Omicron variants of the RBD region using PONDR^®^ VLXT.

Variant	No. of residues disordered	Overall percent disordered	Predicted disorder segment	Number disordered regions
Wuhan-RBD	14	6.28	[1]–[6] [150]–[157]	2
Delta-RBD	9	4.04	[1]–[6] [153]–[155]	2
Omicron-RBD	6	2.62	[1]–[6]	1
1058_RBD(B.1.1)	12	5.38	[1]–[6] [150]–[155]	2
1003_RBD(BA.1.5)	6	2.69	[1]–[6]	1
1044_RBD(BA.2)	8	3.59	[1]–[6] [91]–[92]	2
1015_RBD(BA.2.10)	8	3.59	[1]–[6] [91]–[92]	2
1017_RBD(BA.2.37)	8	3.59	[1]–[6] [91]–[92]	2
1092_RBD(BA.2.38)	8	3.59	[1]–[6] [91]–[92]	2
1125_RBD(BA.2.74)	8	3.59	[1]–[6] [91]–[92]	2
1134_RBD(BA.2.75)	8	3.59	[1]–[6] [91]–[92]	2
1129_RBD(BA.4)	8	3.59	[1]–[6] [91]–[92]	2
1146_RBD(BA.5.2)	8	3.59	[1]–[6] [91]–[92]	2
1142_RBD(XBB)	8	3.59	[1]–[6] [91]–[92]	2
1145_RBD(XBB.3)	8	3.59	[1]–[6] [91]–[92]	2
1148_RBD(XBB.2.3.7)	12	12.11	[1]–[6] [85]–[95] [150]–[159]	3
1169_RBD(XBB.1.16)	30	13.45	[1]–[6] [91]–[92] [147]–[168]	3
1176_RBD(XBB.1)	30	13.45	[1]–[6] [91]–[92] [147]–[168]	3
1194_RBD(BA.3)	30	13.45	[1]–[6] [91]–[92] [147]–[168]	3

A protein stability study for the RBD mutations was done using I-MUTANT, SIFT, and POLYPHEN-2 tools (Table 6). All amino acid changes in the Delta variant were responsible for decreased stability, were found to have a mutation that is tolerated, and had a benign score. However, all mutations in Omicron-RBD decrease stability and are tolerated except for the mutation Y505H, which affects the protein function and increases the disease vulnerability. In addition, the mutations S371L, S373P, S375F, K417N, E484A, G496S, and Y505H in the reference Omicron have a damaging effect on protein structure and function. Mutations unique to different Omicron sub-variants and recombinants such as R346T, T376A, D404N, D405N, R408S, T457K, E478V, and F486P pose a decreased stability and affect the structure and function of the protein leading to increased disease susceptibility and evasion of neutralizing antibody-mediated host response. However, with so many changes in the protein structure and function, it might also be responsible for the decrease in the severity of the disease itself.

**Table 6:** A protein stability study for the RBD mutations was done using I-MUTANT, SIFT, and POLYPHEN-2 tools.

Mutatant	AA change	I-Mutant	SIFT	PolyPhen-2
Delta (B.1.617.2)				
	L452R	Decrease stability −1.04	Tolerated (0.50)	Benign (score 0.017)
	T478K	Decrease stability −0.09	Tolerated (0.92)	Benign (score 0.000)
OMICRON_REFERENCE 1003_RBD(BA.1.5)				
	G339D	Decrease stability −2.06	Tolerated (1.00)	Benign (score 0.002)
	S371L	Decrease stability −0.03	Tolerated (0.39)	Probably damaging (0.997)
	S373P	Decrease stability −1.26	Tolerated (0.29)	Probably damaging (0.956)
	S375F	Decrease stability −0.40	Affect protein function (0.03)	Probably damaging (score 1.0)
	K417N	Decrease stability −0.33	Tolerated (0.33)	Possibly damaging (score 0.747)
	N440K	Decrease stability −0.86	Tolerated (0.51)	Benign (score 0.011)
	G446S	Decrease stability −1.21	Tolerated (0.97)	Benign (score 0.002)
	S477N	**Increase stability 0.01**	Tolerated (0.12)	Benign (score 0.001)
	T478K	Decrease stability −0.09	Tolerated (1.00)	Benign (score 0.00)
	E484A	Decrease stability −1.12	Tolerated (0.35)	Possibly damaging (score 0.484)
	Q493R	Decrease stability −0.38	Tolerated (0.07)	Benign (score 0.00)
	G496S	Decrease stability −1.21	Tolerated (0.19)	Probably damaging (score 0.986)
	Q498R	Decrease stability −1.15	Tolerated (0.44)	Benign (score 0.03)
	N501Y	**Increase stability −0.34**	Affect protein function (0.04)	Benign (score 0.441)
	Y505H	Decrease stability −1.20	Affect protein function (0.00)	Probably damaging (score of 1.00)
1058_RBD(B.1.1)				
	E484A	Decrease stability −0.48	Tolerated (0.41)	Possibly damaging (0.523)
	Y505H	Decrease stability −1.20	Affect protein function (0.00)	Probably damaging (1.000)
1044_RBD(BA.2) 1015_RBD(BA.2.10) 1017_RBD(BA.2.37) 1092_RBD(BA.2.38)				
	S371F	Decrease stability −0.07	Tolerated (1.00)	Probably damaging (score of 0.996)
	T376A	Decrease stability −1.36	Tolerated (0.08)	Probably damaging (score of 0.996)
	D405N	Decrease stability −1.58	Tolerated (0.61)	Probably damaging (score of 0.911)
	R408S	Decrease stability −2.20	Tolerated (0.82)	Probably damaging (score of 1)
	K417N	Decrease stability −0.33	Tolerated (0.33)	Possibly damaging (0.747)
1125_RBD(BA.2.74)				
	R346T	Decrease stability −1.86	Tolerated (0.46)	Probably damaging (score of 0.957)
	L452M	Decrease stability −0.49	Tolerated (0.23)	Probably damaging (score of 0.957)
	Q493R	Decrease stability −0.38	Tolerated (0.07)	Benign (score 0.00)
1134_RBD(BA.2.75)				
	G339H	Decrease stability −2.34	Tolerated (0.13)	Probably damaging (score of 0.964)
	G446S	Decrease stability −1.21	Tolerated (0.97)	Benign (score 0.02)
	N460K	Decrease stability −1.65	Tolerated (1.00)	Probably damaging (score of 0.957)
1129_RBD(BA.4)				
	L452R	Decrease stability −1.40	Tolerated (0.20)	Benign (score 0.017)
	F486V	Decrease stability −3.10	Tolerated (0.14)	Benign (score 0.181)
1146_RBD(BA.5.2)				
	R346T	Decrease stability −1.86	Tolerated (0.61)	Probably damaging (score of 0.957)
	L452R	Decrease stability −1.40	Tolerated (0.20)	Benign (score 0.017)
	F486V	Decrease stability −3.10	Tolerated (0.14)	Benign (score 0.181)
1142_RBD(XBB) 1145_RBD(XBB.3)				
	G339H	Decrease stability −2.34	Tolerated (0.13)	Probably damaging (score of 0.964)
	R346T	Decrease stability −1.86	Tolerated (0.61)	Probably damaging (score of 0.957)
	L368I	Decrease stability −0.61	Affect protein function (0.02)	Probably damaging (score of 0.860)
	S371F	Decrease stability −0.07	Tolerated (1.0)	Probably damaging (score of 0.999)
	K417N	Decrease stability −0.33	Tolerated (0.33)	Probably damaging (score of 0.747)
	V445P	Decrease stability −1.93	Tolerated (0.18)	Benign (score 0.146)
	G446S	Decrease stability −1.21	Tolerated (0.97)	Benign (score 0.002)
	N460K	Decrease stability −1.65	Tolerated (1.00)	Benign (score 0.001)
	F486S	Decrease stability −3.28	Affect protein function (0.02)	Possibly damaging (0.941)
	F490S	Decrease stability −2.99	Affect protein function (0.01)	Benign (score 0.420)
1169(XBB.1.16)				
	E478V	Increase stability 0.18	Tolerated (0.18)	Benign (score 0.006)
	F486S	Decrease stability −3.28	Affect protein function (0.02)	Possibly damaging (0.941)

### SARS-CoV-2 RBD interaction with host ACE2

**Figure 7:**
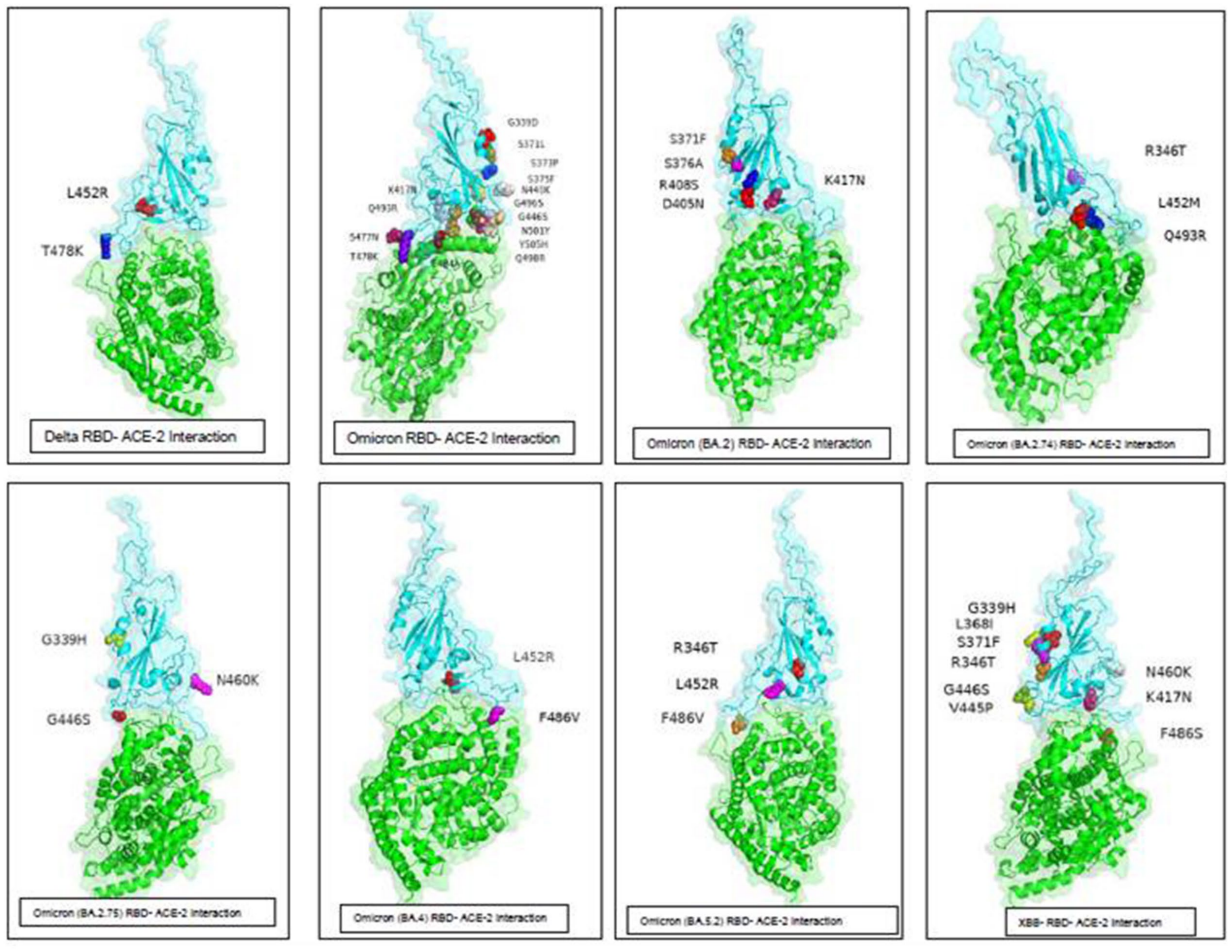
ACE-2-RBD of Omicron variants interaction and important mutations in the RBD region of the different variants of Omicron sequenced from this region.

The spike protein of SARS-CoV-2 is the most important surface protein of the virus, which is primarily responsible for the entry of the virus into the human host mediated by the ACE2 receptor. The RBD-ACE2 receptor complex plays a major role in the entry of the virus into the host cell. Mutations in spike protein have led to the emergence of new variants over the period of time, currently circulating hypermutated Omicron variant has retained the D614G substitution seen in the Delta variant with mutations N440K, T478K, and N501Y in the critical region of RBD–ACE2 receptor interaction. In addition, the mutations K417N, E484A, and Y505H of Omicron affect the antibody response against the spike protein leading to an increased immune escape phenomenon. Understanding the RBD–ACE2 receptor interaction is important because it is responsible for the infectivity of the virus, host response, and range of disease manifestations. As protein–protein interaction tools are simple and require less computing-intensive resources than Molecular Dynamics simulation studies. ClusPro was used to understand the binding affinity of the ACE2 receptor and RBD of the Omicron variants sequenced from this region (Table 7). The molecular structure of RBDs of all different sub-lineages was constructed using the AlphaFold2. RBD structures were then used for docking with the ACE2 receptor. PBD crystal structure of ACE2-SARS-CoV-2 RBD (6M0J) was used to get the ACE2 receptor structure by removing the SARS-CoV-2 RBD part and the receptor was used for the docking study. Docking was done using Delta, Omicron, and Omicron sub-lineages RBD, which were sequenced during this study with the ACE2 receptor (Figure 6). The highest docking score was seen with the Omicron variant suggesting that the mutations in the spike region have made it more responsive for the ACE2 receptor leading to a greater potential for transmission. The lowest docking score was seen for the B.1.1 variant and BA.1 has the highest score, probably making it the most circulating variant worldwide. Among the BA.2 variants, there is a decline in the docking score in BA.2.23, BA.2.38, and BA.2.76 and was a similar score to that of the Delta strain. However, the BA.2.74 and BA.2.75 show a return of high docking scores, making them a candidate for the most circulating strain. However, the docking score of BA.4 is lower, making it less responsive to ACE-2 (see Figure 7).

**Table 7:** Docking analysis of Wuhan-RBD, Delta-RBD, Omicron-RBD, and Omicron variants RBD with ACE2 using ClusPro software.

S. No.	Variant with ACE2	Docking energy
1	WUHAN_RBD	−953.7
2	DELTA_RBD	−868.3
3	OMICRON	−1007.4
4	1015_RBD(BA.2.10)	−975.2
5	1017_RBD(BA.2.37)	−975.2
6	1044_RBD(BA.2)	−975.2
7	1058_RBD(B.1.1)	−912.3
8	1125_RBD(BA.2.74)	−947.5
9	1134_RBD(BA.2.75)	−902.8
10	1145_RBD(XBB.3)	−813.5
11	1142_RBD(XBB)	−813.5
12	1129_RBD(BA.4)	−857.4
13	1003_RBD(BA.1.5)	−1156.8
14	1092_RBD(BA.2.38)	−884.7
15	1146_RBD(BA.5.2)	−953.7
16	1148_RBD(XBB.2.3.7)	−809.8
17	1169_RBD(XBB.1.16)	−823.8
18	1176_RBD(XBB.1)	−823.8
19	1194_RBD(BA.3)	−838.1

## Discussion

The detailed pan-India studies revealed the predominance and emergence of the SARS-CoV-2 virus clades and variants circulating in different parts of India revealed GH and GR as predominant clades along with signature mutations E484Q and N440K appeared first in Maharashtra (24). Another pan-India study conducted revealed spreading patterns across the country (25). Apart from this, the phylodynamic patterns were also deciphered and the predominance of D614G was studied across the country (26).

Spike protein is a 3,821-nucleotide-long ranging from nucleotide (Nt) position 21,563–25,384 with an amino acid (AA) sequence of 1,273 in the genome sequence of SARS-CoV-2. The receptor-binding domain (RBD) region which lies from Nt position 319–541 in the S protein showed a maximum number of mutations. Signature mutations seen in S protein were T19I, G142D, V213G, G339D, S371F, S373P, S375F, T376A, D405N, R408S, K417T, N440K, S477N, T478K, E484A, Q493R, Q498R, N501Y, Y505H, D614G, H655Y, N658S, N679K, P681H, D796Y, Q954H, and N969K. Mutations commonly seen in RBD were G339D, R346T, S371F, S373P, S375F, T376A, D405N, R408S, K417T, N440K, L452M, S477N, T478K, E484A, Q493R, N501Y, and Y505H. BA.1 had a unique mutation T547K in addition to all common mutations of the original Omicron variant (hCoV-19/Botswana/R40B59 BHP 3321001248/2021) in the RBD region. T376A, D405N, and R408S mutation were new and common to all BA.2 variants. Additionally, R346T was seen in emerging BA.2.74 and BA.2.76 variants. However, the BA.2.75 variant did not show this R346T mutation. The emerging BA.4 retained the common T376A, D405N, and R408S mutations of BA.2 along with a new mutation F486V. Signature mutations seen in Orf1 ab of BA.2 were S135R, T842I, G1307S, A1824V, L3027F, T3090I, L3201F, T3255I, and F3677L. The nucleocapsid domain had signature mutations P13L, R203K, G204R, and S413R and deletion of three codons 31–33. XBB variant evolved over time showing T19I, L24S, del25/27, V83A, G142D, del144/144, H146Q, Q183E, V213E, G339H, R346T, L368I, S371F, S373P, S375F, T376A, D405N, R408S, K417N, N440K, V445P, G446S, N460K, S477N, T478K, E484A, F486S, F490S, Q498R, N501Y, Y505H, D614G, H655Y, N679K, P681H, N764K, D796Y, Q954H, and N969K in the spike region (14, 27, 28). XBB.3 and XBB.1.5 variants showed an additional mutation in spike region G252V. XBB.1.16, XBB subvariant with two substitutions in the S protein (E180V, T478R) compared to XBB.1.5. The samples sequenced were from different districts of Madhya Pradesh and showed a predominance of BA.2 and its variants circulating in this region during the third wave. The current study noted a very few BA.1, B.1.1 variants which were from the initial period of the study. The previously predominant Delta strain of the second wave has been replaced by the Omicron variant in this region over a period of Feb to Jul 22. Interestingly, BA.1 is circulating in other of the world, this region showed a predominance of the BA.2 variant. In a recent study, it has been published that BA.2 is approximately 1.5 and 4.2 times more than infectious as BA.1 and Delta, respectively. This study showed a continuous shift of sub-lineages of BA.2, and further emergence of BA.4 is seen among the recent samples. The emergence of another wave of SARS-CoV-2 infection was seen with recombinant variant XBB.1.5 in the late 2022 year followed by XBB.1.16 in the month of Feb 2023, around the globe. XBB variants have shown increased ACE receptor binding and higher immune evasion due to the substitution of F486S amino acid in the glycoprotein region of the spike. Previous studies have shown that the hypermutated, Omicron variant is milder than the Delta variant but is more infectious and has a tendency to evade the antibody response, leading to the serious concern of SARS-CoV-2 infection in previously infected and immunized patients. Studies on molecular changes in spike protein have shown evidence of reduced activity of neutralizing monoclonal antibodies. Mutations in the RBD region of spike protein play a crucial role in the infectivity affecting the human ACE2 receptor–RBD interactions. Previous studies have shown that N440K, T478K, and N501Y mutations are important determinants for specific recognition of SARS-CoV-2 viral RBD by human ACE2 receptor. Numerous mutations have been noted in the RBD region of the Omicron variant when compared to the Delta variant, which suggests that RBD can be a potential candidate for the sub-unit vaccine for booster immunization providing a robust neutralizing antibody response. A protein with less than 40 instability index (II) predicted stable protein, whereas a value of more than 40 predicts unstable protein. Selected sub-variant scores remained between 23.07 and 31.31, indicating the greater stability of all SARS-CoV-2 RBD regions except for the variant BA.2.74 whose II was higher than the Delta-RBD region, making it vulnerable to mutations. Presently circulating recombinants-XBB.1, XBB.1.16, and XBB.3 have a higher II raising the possibility of emerging variants in the near future. The aliphatic (alanine, leucine valine, and isoleucine) chains occupy the relative volume and are defined as the aliphatic index of a protein and this is regarded positive factor for the increase in thermostability of protein. A higher protein’s aliphatic index indicates that the protein is thermostable across a wide range of temperatures. This aliphatic index of RBD protein of all Omicron variants and recombinants is higher than the Delta and Wuhan-Hu-1, making them more thermostable. Hydropathicity is the degree to which amino acids in a protein sequence are hydrophobic or hydrophilic, and the Grand Average of Hydropathicity Index (GRAVY) is used to represent the hydrophobicity value of a peptide, and a score below 0 is considered a hydrophilic protein. This value is consistent over all the variants of SARS-CoV-2. This evaluation of the secondary structure of RBD of selected Omicron variants, which were sequenced in this study was done, it was noted that there was an increase in alpha-helix structure among most Omicron sub-variants, however, there was noted a deviation in some BA.2 sub-lineages and B.1.1 variant(1058_RBD). RBD of the B.1.1 variant showed a similar secondary structure to the Delta variant with 6.73% alpha-helix and 69.51% random coil. Over a period of time there is a change noted in evolving and circulating variants, BA.2.74 showed a sharp dip in the percentage of the alpha-helix (2.24%) when compared with the Delta strain, however, maintaining the random coil to 72.65% and the extended strand to 25.11%. RBD of recombinant variants (XBB, XBB.1, and XBB.3) and BA.5.2 also showed a change in the alpha-helix (4.93%) similar to Delta but the random coil maintained by 72.2%. XBB.2.3.7 variant showed a further dip in the alpha-helix (3.59%) lower than that of the Delta with a dip in random coil (65.92%) as well. This continuous change in the structure of these variants at the important receptor-binding site of the virus possibly explains the evasion of host immune response, the difference in the severity of the disease, and the transmissibility. Recombinants have shown a further decrease in the docking score, making them less responsive to the ACE-2. These changes might lead to less responsiveness and less spread of these recombinants. Further molecular dynamics simulation studies are required to look into the molecular interaction of the receptor–ligand complex and its effect on molecular mechanism in terms of virus entry and its role in the pathogenicity of the disease *per se*. Several groups also have discussed the SARS-CoV-2 (Omicron) genome dynamics and its potential introduction roles in detail. These studies also identified country-wide unique substitutions across the genome. These initial introductions at genome levels were contributing to infectious SARS-CoV-2 among communities (29–32). These will help in understanding the disease mechanism, host-immune evasion, and also in developing therapeutics.

## Data availability statement

The original contributions presented in the study are included in the article/supplementary material, further inquiries can be directed to the corresponding author.

## Ethics statement

The study protocol was approved by DRDE-Institutional Biosafety Committee vide No. IBSC/VIRO-02/2020/PKD. Experiments in this study were conducted according to biosafety and regulatory guidelines. Ethical clearance was obtained from Vidhya Ethics Clearance vide No. VCH/VEC/Feb-2023/01. The waiver from written Informed consent and ethical approval for the study was obtained from Vidhya Ethics Committee Gwalior M.P. India vide No. VCH/VEC/Feb-2023/01.

## Author contributions

SD: Investigation, Methodology, Writing – original draft, Formal analysis. PY: Methodology, Writing – original draft. SS: Methodology, Writing – original draft, Formal analysis, Investigation, Writing – review & editing. EG: Methodology, Writing – original draft. RY: Methodology, Writing – original draft. PD: Conceptualization, Formal analysis, Funding acquisition, Supervision, Validation, Resources, Writing – review & editing. MP: Funding acquisition, Project administration, Resources, Supervision, Writing – review & editing.

## Footnotes

^1^ https://clades.nextstrain.org/tree
